# Changes of Cell Biochemical States Are Revealed in Protein Homomeric Complex Dynamics

**DOI:** 10.1016/j.cell.2018.09.050

**Published:** 2018-11-15

**Authors:** Bram Stynen, Diala Abd-Rabbo, Jacqueline Kowarzyk, Leonor Miller-Fleming, Simran Kaur Aulakh, Philippe Garneau, Markus Ralser, Stephen W. Michnick

**Affiliations:** 1 Département de Biochimie, Université de Montréal, C.P. 6128, Succursale Centre-ville, Montréal, QC H3C 3J7, Canada; 2 Department of Biochemistry, University of Cambridge, 80 Tennis Court Road, Cambridge CB2 1GA, UK; 3 Molecular Biology of Metabolism Laboratory, The Francis Crick Institute, 1 Midland Road, London NW1 1AT, UK; 4 Department of Biochemistry, Charité University Medicine, Berlin, Germany; 5 Centre Robert-Cedergren, Bio-Informatique et Génomique, Université de Montréal, C.P. 6128, Succursale centre-ville, Montréal, QC H3C 3J7, Canada

**Keywords:** large-scale screen, protein-protein interactions, metformin, rapamycin, aging, iron homeostasis

## Abstract

We report here a simple and global strategy to map out gene functions and target pathways of drugs, toxins, or other small molecules based on “homomer dynamics” protein-fragment complementation assays (*hd*PCA). *hd*PCA measures changes in self-association (homomerization) of over 3,500 yeast proteins in yeast grown under different conditions. *hd*PCA complements genetic interaction measurements while eliminating the confounding effects of gene ablation. We demonstrate that *hd*PCA accurately predicts the effects of two longevity and health span-affecting drugs, the immunosuppressant rapamycin and the type 2 diabetes drug metformin, on cellular pathways. We also discovered an unsuspected global cellular response to metformin that resembles iron deficiency and includes a change in protein-bound iron levels. This discovery opens a new avenue to investigate molecular mechanisms for the prevention or treatment of diabetes, cancers, and other chronic diseases of aging.

## Highlights

• *hd*PCA reveals dynamics of protein homeostasis
• *hd*PCA uncovers the responses of most biochemical pathways to cell perturbations
• An *hd*PCA screen reveals that metformin induces an iron deficiency-like state
• *hd*PCA can be applied to mapping out biochemical pathways and networks

## Graphical Abstract

**Figure:**
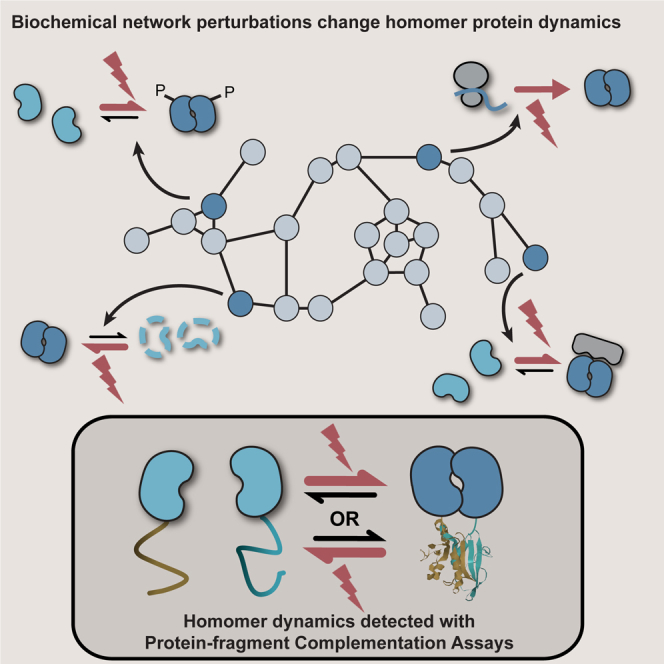

## Teaser

Assessment of the global effects of small molecules on protein self-association in yeast provides insights into target pathways and mechanisms of action.

## Introduction

**Figure 1:**
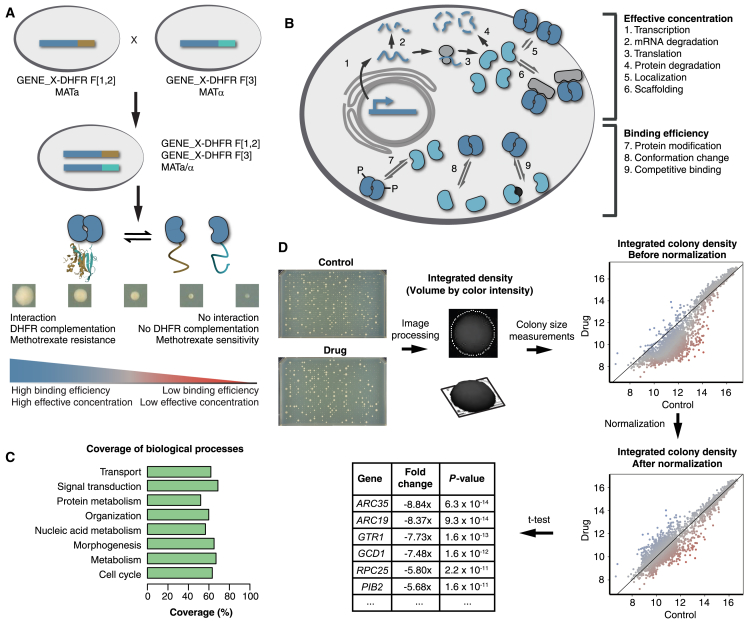
A Homomer Dynamics DHFR PCA for the Detection of the Condition-Dependent States of Proteins (A) A library of homomer dynamics DHFR PCA (*hd*PCA) strains is created by mating two strains, each containing an open reading frame (ORF) of interest tagged with one of the two complementary fragments of murine dihydrofolate reductase (mDHFR, brown and light blue). Upon interaction of two molecules of the same protein, the two fragments of mDHFR fold and reconstitute into a functional enzyme. This reconstitution quantitatively correlates with growth in the presence of methotrexate (Levy et al., 2014) and is determined by effective concentration and binding efficiency. (B) The degree of homomerization (self-association) of a protein is the result of different factors, some of which influence the effective concentration (top), whereas others influence binding efficiency (bottom). (C) Coverage of gene ontology (GO) biological processes in the *hd*PCA, with coverage determined by the percentage of proteins associated with a GO Super-Slim biological process, that have been screened in the *hd*PCA. GO Super-Slim biological processes were obtained by manually condensing the standard terms in the GO Slim (available at https://www.yeastgenome.org/download-data/curation, as of July 2017) into eight GO global terms. (D) Workflow of the *hd*PCA.

Linking the effects of environmental chemicals, nutrients, or drugs to specific cellular functions requires a screening strategy that can probe all or most biochemical processes. Ideally, such a strategy reports on both passive and indirect (e.g., protein or mRNA turnover) and active (e.g., post-translational modification) effects on a pathway. High-throughput methods such as mRNA and protein abundance profiling can inform about states of pathways. However, they measure the effects of perturbations indirectly, on passive processes. Large-scale screening of eukaryotic biochemical pathways can be achieved in the budding yeast *Saccharomyces cerevisiae* by measuring synthetic chemical-genetic interactions (chemical epistasis) to identify target pathways or specific proteins (Giaever et al., 2004, Parsons et al., 2006). Recently, a combination of synthetic genetic array technology with high-content screening has generated the first flux network for budding yeast in which protein abundance and localization are tracked in response to a chemical over time (Chong et al., 2015). We have developed a simple alternative to chemical epistasis or high-content screens in yeast that does not require gene knockouts and can capture the integrated passive and active effects of cellular perturbation on many reporter proteins. In our strategy, we measure condition-dependent changes in homo-oligomerization of proteins in living yeast using a homomer dynamics protein-fragment complementation assay (*hd*PCA). This method is based on protein-protein interaction-driven folding and reconstitution of a methotrexate-resistant murine dihydrofolate reductase (mDHFR) from complementary N- and C-terminal fragments. The coding sequences of the complementary fragments (F[1,2] and F[3]) of mDHFR are integrated into the genome, 3′ to the open reading frames of genes of interest (Figure 1A). In *hd*PCA, mDHFR F[1,2] and F[3] are integrated into the individual alleles of a single gene in the mating-type strains BY4741 *MAT*a and BY4742 *MAT*α, respectively. Mating of these two strains results in two copies of the same gene tagged with complementary mDHFR fragments. Homo-oligomerization of the tagged protein results in reconstitution of mDHFR activity. Cells with the reconstituted mDHFR can divide in methotrexate, which has no effect on the mDHFR mutant but inhibits the yeast DHFR, resulting in colonies whose size is proportional to the number of homomeric complexes per cell (Remy and Michnick, 1999).

Applications of PCA screens have been reported to measure the effects of drugs on specific pathways or to map out specific mechanisms of action of drugs (Freschi et al., 2013, MacDonald et al., 2006, Rochette et al., 2014, Schlecht et al., 2012). For instance, we have previously used a series of heteromeric fluorescent protein reporter PCAs to identify potential anticancer agents in mammalian cells (MacDonald et al., 2006). The *hd*PCA differs from these strategies because the focus is shifted from protein-protein interactions to individual protein states. Any change in the number of homomeric complexes results from the integrated passive and/or active effects of a molecule acting on a pathway in which a reporter protein participates (Figure 1B). The change in the number of homomeric complexes will then affect the colony growth of the corresponding *hd*PCA strain. Examples of protein properties on which the mDHFR PCA can report include a change in binding affinity, post-translational modifications, localization, and abundance (Filteau et al., 2015, Lev et al., 2013, Levy et al., 2014, Remy et al., 1999).

Here we report the results of *hd*PCA screens of two small molecules, rapamycin, an immunosuppressive and antiproliferative drug that inhibits a very specific enzyme target, and metformin, an antiglycemic diabetes drug that has no established known direct target, presenting contrasting molecular features to test the performance of *hd*PCA.

## Results

### *hd*PCA Dynamics Provide Reporters for Virtually All Cellular Processes

Homomeric interactions are quite common; for instance, at least 50% of proteins in the bacterium *Mycoplasma pneumoniae* form homomers, and 47% (41,618 of 88,739) of high-resolution structures of protein complexes are homomeric (Krissinel and Henrick, 2007, Kühner et al., 2009). Strong binding affinities are not essential to detect changes in homomerization in the mDHFR PCA screen. Changes in the *hd*PCA signal of proteins with low self-binding affinity or low abundance can still be detected with statistical significance (Tables S1 and S2). The 3,504 *hd*PCA reporters cover ∼60% of the yeast proteome equally distributed among the major categories of cellular processes, and, thus, we have reporters for practically all cellular processes (Figure 1C). The virtue of the *hd*PCA screening strategy is its simplicity and economy. The 3,504 diploid *hd*PCA strains can be arrayed at high density on just three agar plates (1,536 strains per plate), and an entire screen with replicates can be performed with only a dozen plates (Figure 1D; STAR Methods).

*hd*PCA strains are spotted as four replicates on plates containing control or test medium. Pictures of the plates are taken over 2 weeks. Growth is scored as integrated pixel density, with final values obtained after two rounds of normalization, one to correct for plate-to-plate variability (quantile normalization) and the other to correct for the basal effect of the drug on growth (local regression [LOESS] normalization). Finally, a statistically significant growth difference is determined with a regularized t test comparing means of the replicates under control and drug conditions (Baldi and Long, 2001).

### *hd*PCA Measures Active and Passive Changes of Proteins Modulated by Rapamycin

To assess the performance of the *hd*PCA screen, we chose the immunosuppressant drug rapamycin as a test perturbation. We chose this molecule because it has well-defined and distinct target receptors (the target of rapamycin [TOR] complex) and known downstream affected cellular processes; like metformin, it has potential anticancer and anti-aging properties; and there exist sources of large-scale measurements of rapamycin on mRNA and protein homeostasis, active processes, and chemical-genetic interactions (Anisimov et al., 2011). These allowed us to test the hypothesis that *hd*PCA can capture the effects of a molecule on a reporter protein and, by extension, on a given cellular process in which the reporter protein is implicated.

**Figure 2:**
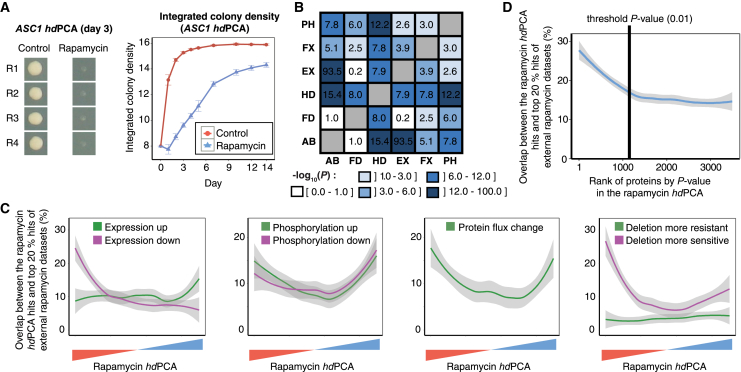
Optimization and Validation of *hd*PCA with Rapamycin Data (A) Growth of a strain containing *ASC1-DHFR-F[1,2]* and *ASC1-DHFR-F[3]* during the course of an *hd*PCA screen with rapamycin. Four replicates (R1 to R4) were tested. Error bars indicate SD. (B) Correlations among different large-scale datasets comparing control versus rapamycin-treated yeast cells. Datasets are compared with each other for significant overlap of genes or proteins affected by rapamycin. Values correspond to –log_10_(*P*), where *P* is the p value of the hypergeometric test that compares significance of overlap between datasets. AB, protein abundance; FD, fitness of deletion strains; HD, *hd*PCA; EX, mRNA expression; FX, protein flux; PH, phosphoproteomics. (C) LOESS curves indicating the running average overlap between rapamycin *hd*PCA hits, ordered from most reduced to most increased *hd*PCA signal in the presence of rapamycin, and the top 20% hits of rapamycin data focused on expression, phosphorylation, protein flux, and deletion strain sensitivity. (D) A LOESS curve indicating the average overlap between *hd*PCA hits, ranked by p value, and the top 20% hits of five external rapamycin datasets (deletion screens, mRNA expression data, phosphoproteomics data, protein abundance data, and protein flux data). The enrichment found in top-ranked *hd*PCA hits falls to background levels close to the threshold p value of 0.01, which was chosen as a threshold value in further experiments. See also Tables S1 and S3.

We performed the *hd*PCA screen with strain arrays grown in the presence or absence of 2 nM rapamycin. As an example of a response to the addition of rapamycin, the strain expressing the *hd*PCA constructs for the protein Asc1 (*ASC1-DHFR-F[1,2]* and *ASC1-DHFR-F[3]*) grows significantly better in the absence of rapamycin (Figure 2A). This likely reflects a change in the state of Asc1. Its human ortholog, the G-protein subunit RACK1, regulates a glutamate-gated ion channel in a homodimerized form (Thornton et al., 2004) and is regulated by rapamycin through changes in both mRNA and protein levels (Erbil et al., 2016, Loreni et al., 2005). Our *hd*PCA data suggest that the Asc1 protein, like RACK1, forms a homodimer (Yatime et al., 2011), an interaction that is regulated by rapamycin (and the TOR pathway), both directly and indirectly.

Large-scale screens that measure individual properties of a gene, its interactions, or its products are notoriously orthogonal to each other, showing little overlap in changes of individual quantities (Ideker and Krogan, 2012). If *hd*PCA truly captures the integrated effects of changes to a protein’s fate following a change of cellular conditions, then we should expect significant correlations between *hd*PCA reporter responses and other large-scale data. We assessed how well the results of the rapamycin *hd*PCA reflect the integrated effects of the drug (Table S1) by comparing the rapamycin-induced changes in *hd*PCA signal to changes in protein and mRNA abundance, post-translational modifications (phosphoproteomics) and protein localization (GFP-based protein flux study), and rapamycin-gene epistasis (see STAR Methods for details regarding the datasets). Based on the degree of overlap of protein hits between datasets, *hd*PCA agrees better with the other datasets than any other dataset among themselves except for mRNA with protein abundance (Figure 2B). This observation implies that *hd*PCA incorporates combinations of passive and active protein changes. Positive *hd*PCA data correlate specifically with increases in gene expression, whereas negative *hd*PCA data correlate with reduced expression (Figure 2C). This direction-dependent correlation does not exist for phosphorylation status, whereas, for deletion sets, a reduced *hd*PCA signal correlates more strongly with deletion strains that increase sensitivity to rapamycin (Figure 2C).

Among the top 10 protein hits with reduced signal in the rapamycin *hd*PCA (Table S1), two (Arc35 and Arc19) are involved in endocytosis, one (Gcd1) in translation, and one (Rpc25) in RNA polymerase III activity, all processes that are stimulated by the TOR pathway or repressed by rapamycin (Barbet et al., 1996, MacGurn et al., 2011, Oficjalska-Pham et al., 2006). Two other proteins (Gtr1 and Pib2) are known to activate the TOR pathway (Stracka et al., 2014, Varlakhanova et al., 2017), one (Uba4) is possibly positively regulated by TOR (Rubio-Texeira, 2007), and one (the V-ATPase complex subunit Vma7) is possibly negatively regulated by TOR (Wilms et al., 2017). The last protein, Erg26, is a member of the ergosterol pathway with no obvious link to the TOR pathway in yeast. Among the top 10 hits with an increase in *hd*PCA signal in the presence of rapamycin (Table S2), three (Hxt5, Vps27, and Gdh3) are positively regulated by rapamycin (Beck et al., 1999, Huang et al., 2004, Kingsbury and Cardenas, 2016). Another top 10 hit is Fpr1 (FKBP12), the primary receptor of rapamycin, which, as a complex, binds to and inhibits TOR activity (Koltin et al., 1991). Among the three top 10 hits without a clear function is YGR201C, whose only interaction partner, Slm4, is a member of the exit from rapamycin-induced growth arrest (EGO) complex that regulates exit from rapamycin-induced growth arrest (Yu et al., 2008).

Overall, a reduced *hd*PCA signal appears to correlate more with reduced protein activity, whereas an increased *hd*PCA signal seems to be more associated with increased protein activity. This observation is in line with a systematic literature survey, where we found that 91% (124 of 136) of homodimers were more active than monomers. For proteins with a clear form of regulation of self-association, 90% of proteins were more active in the homodimeric than monomeric state (27 of 30; Table S3).

The overlap between rapamycin *hd*PCA hits (affected strains) and hits in other rapamycin large-scale studies was used to determine a criterion for a significance cutoff of differences in colony size between control- and drug-treated strains. At p > 0.01, the overlap between rapamycin *hd*PCA data and other datasets reached a plateau and, hence, was chosen as a cutoff criterion (Figure 2D). The significant results include 124 proteins that are essential for yeast and that are difficult to study in deletion screens.

### The *hd*PCA Reveals Pleiotropic Cellular Effects of the Antidiabetic Drug Metformin

Metformin is currently one of the most prescribed forms of non-insulin therapy among type 2 diabetes (T2D) patients. Recently, potential novel health benefits of metformin have been revealed that could improve the human health span. These include retrospective studies in humans and increased lifespan in mice that have motivated a prospective clinical study of metformin (Barzilai et al., 2016, Martin-Montalvo et al., 2013, Pryor and Cabreiro, 2015, UK Prospective Diabetes Study (UKPDS) Group, 1998). No specific metformin-binding target has been identified to date, and it likely acts on multiple targets (Madiraju et al., 2014, Owen et al., 2000).

Confounding efforts to understand the effects of metformin is that many *in vitro* and animal studies of metformin have been performed at as high as millimolar concentrations, 100-fold greater than the clinically relevant range of 10 μM (Madiraju et al., 2018). We found that effective concentrations to observe a growth effect of metformin were in the range of 50 mM, and we used this concentration in our studies. This concentration obviously exceeds the physiological blood concentration by roughly 1,000-fold (Madiraju et al., 2018) and a concentration 50-fold higher than what is generally used in human cell lines. However, it is well documented that yeasts are highly resistant to a variety of drugs because of their extremely efficient ATP-binding cassette (ABC) and major facilitator superfamily (MFS) multidrug efflux pumps (Sá-Correia et al., 2009). For example, methotrexate, used in this study, requires 2,000 times the concentration used in human cell lines while preserving the same mechanism of action. Consequently, we expect that the effective intracellular concentrations of metformin in our experiments would be within the clinically relevant range.

**Figure S1:**
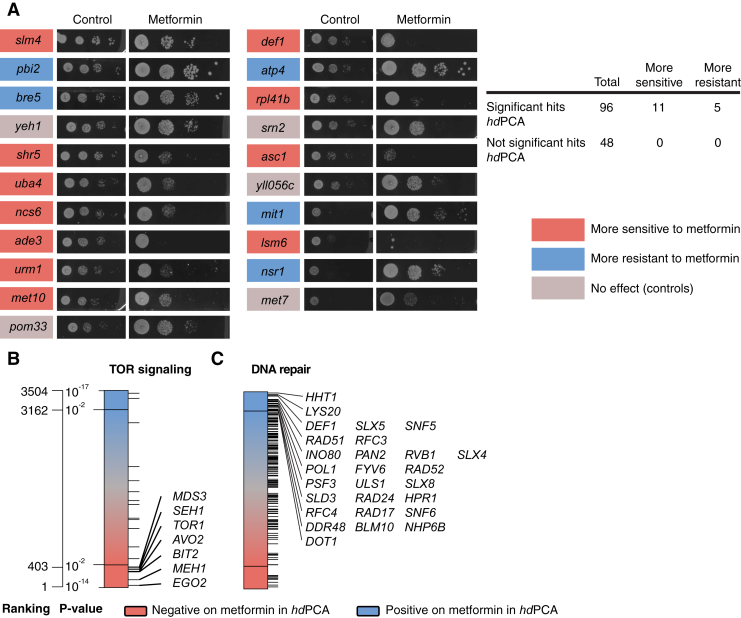
Metformin *hd*PCA Identifies Proteins Involved in Metformin Resistance, in TOR Signaling, and in DNA Repair, Related to Figure 3 (A–C) (A) A deletion miniscreen confirms contribution of significant hits from the metformin *hd*PCA in metformin resistance. Significant hits from the metformin *hd*PCA screen were tested for their involvement in metformin sensitivity by testing their growth in the presence of the drug. Ninety-six deletion strains from significant hits were tested together with 48 negative control strains. No strains from the negative control set were affecting metformin sensitivity. Pictures for the control condition were taken after 2 days, for metformin after 5 days. A chi-square test of independence (metformin versus control) confirms that metformin *hd*PCA hits are more likely to modulate resistance (increased or reduced) to metformin compared to the controls (p < 0.01). Metformin *hd*PCA data on proteins involved in TOR signaling (B) and proteins involved in DNA repair (C).

The *hd*PCA metformin screen revealed 342 and 403 proteins with an increased or reduced signal, respectively (Table S2). Protein hits from the screen were more likely to modulate yeast sensitivity to metformin than proteins whose *hd*PCA signal was not significantly changed by the drug, as determined with a targeted deletion screen (Figure S1A; chi-square test: p < 0.01). There was no correlation between sensitivity and the direction of the *hd*PCA signal, although this might have been caused by the limited size of the screen (144 strains).

**Figure 3:**
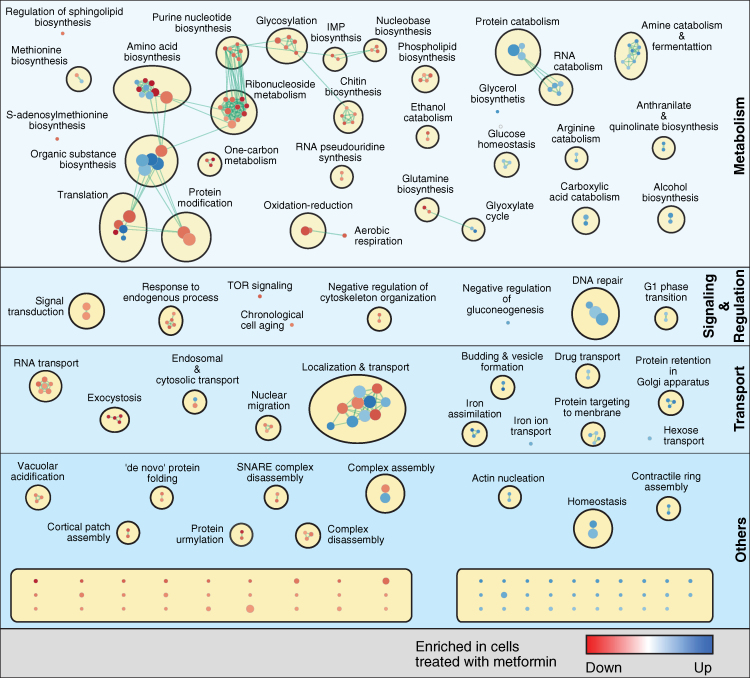
Enrichment Map of GO Biological Processes in the Metformin *hd*PCA The map displays the enriched GO terms in metformin versus control (blue) and those that are enriched in control versus metformin (red). GO terms that have associated genes in common are linked with an edge. The edge width is proportional to the overlap between the linked GO terms. GO terms closer to each other in space are more functionally related than those further away from each other in space and are clustered together. Ungrouped processes not mentioned in the main text are found at the bottom (unnamed; see Table S4 for more details). The figure was generated using the Enrichment Map (GO terms cutoff, 0.05; similarity measure, Jaccard coefficient with default settings) and AutoAnnotate (default settings) plug-ins. See also Figure S1 and Tables S2, S4, S5, S6, and S7.

We used GOstats to identify biological processes that are overrepresented in proteins showing an increased signal on metformin and a control, respectively. The enriched Gene Ontology (GO) terms (p < 0.05) are illustrated as a network of biological processes that are organized in space according to their mutual overlap and clustered based on their relationships (Falcon and Gentleman, 2007, Kucera et al., 2016, Merico et al., 2010). The GO biological processes affected by metformin were classified into four groups (metabolism, signaling and regulation, transport, and other processes); enriched or depleted molecular function and cellular component GO terms were also tabulated (Figure 3; Table S4).

We next investigated the relationship between our observations and potential beneficial effects of metformin for T2D and for prevention of different cancers (Kasznicki et al., 2014). We assessed the overlap between metformin *hd*PCA hits and human homologs containing SNPs associated with T2D and with different types of cancer identified by genome-wide association studies (GWASs) that are annotated in the GWAS Catalog database (Barbet et al., 1996, MacGurn et al., 2011, Oficjalska-Pham et al., 2006, Welter et al., 2014; Table S5). We found a significant overlap between our hits and homologous genes associated with T2D and prostate cancer (p = 0.01 and 0.02, respectively; hypergeometric test; Tables S6 and S7). We found 11 genes in common between our hits and homologs associated with T2D, including the human homolog of *COT1*, *SLC30A8*, which encodes a zinc transporter in the secretory vesicles of pancreatic β cells and was found to be associated with T2D in 12 of 42 GWASs (Table S6). We also found 15 genes whose human homologs are associated with prostate cancer (Sung, 1994, Wintersberger et al., 1995; Table S7).

### *hd*PCA Predicts Effects of Metformin on Energy Metabolism

**Figure 4:**
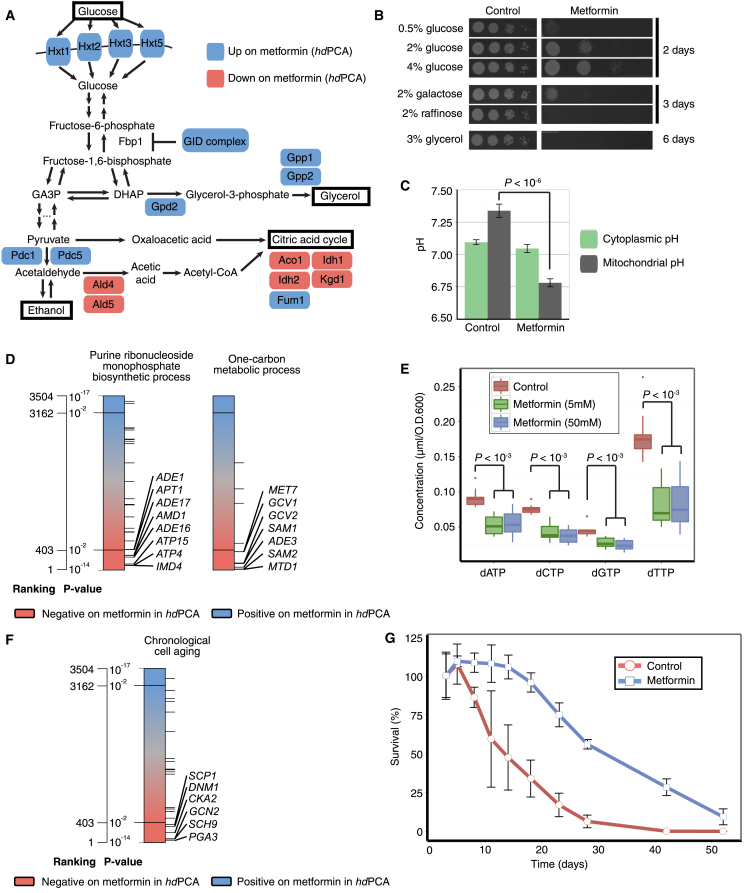
Metformin Influences a Range of Cellular Processes (A) Metformin increases (blue) and decreases (pink) the *hd*PCA signal of proteins involved in glucose uptake and catabolism. (B) Sensitivity to metformin changes with carbon source. Pictures were taken on different days to account for the basal effect of carbon source on growth. (C) The mitochondrial membrane proton gradient reverses after prolonged metformin treatment (7 hr). The pH level was determined using a pH-dependent fluorescent protein (pHluorin) with or without a mitochondrial localization sequence. Error bars indicate the SD (n = 12). (D) The *hd*PCA signals for proteins involved in purine ribonucleoside biosynthesis and one-carbon metabolism are reduced in the presence of metformin. Each horizontal line represents one protein member of the biological process. (E) Metformin at 5 mM and 50 mM reduces the concentration of dNTPs. Concentrations were determined by mass spectrometric analysis of the individual dNTPs. Error bars indicate SD (n = 12). (F) *hd*PCA results for members of the GO term “chronological cell aging.” (G) Metformin prolongs the chronological lifespan. Three cultures were incubated in the presence of metformin and three in its absence. Cell survival was tracked over time by counting the colony-forming units at regular time points. The error bars correspond to the SD of three replicates of one qualitatively representative experiment. GID, glucose-induced degradation; GA3P, glyceraldehyde-3-phosphate; DHAP, dihydroxyacetone phosphate.

Metformin effects on energy metabolism play a large role in its antidiabetic activity. Metformin stimulates glucose uptake, reduces gluconeogenesis and cellular respiratory capacity, and increases concentrations of glycerol and lactic acid (reviewed in Foretz et al., 2014). The *hd*PCA screen showed an increased signal for the major glucose transporters for proteins involved in breakdown of gluconeogenic enzymes and for enzymes involved in glycerol and ethanol production (Figures 3 and 4A). In contrast, the *hd*PCA signals of members of the citric acid cycle and oxidative phosphorylation were reduced. These results are consistent with observations of increased glucose uptake and glycerol production in yeast despite its lack of a structurally conserved mitochondrial complex I, the most cited potential target of metformin (Borklu-Yucel et al., 2015). Sensitivity to metformin follows the degree to which yeast depends on respiration. Higher amounts of the fermentable sugar glucose reduce the effect of metformin on yeast growth, whereas carbon source conditions that rely partially (galactose and low glucose) or completely (glycerol) on respiration increase sensitivity to metformin (Figure 4B). Furthermore, the proton gradient across the mitochondrial membrane is reversed after prolonged metformin exposure (Figure 4C), indicating interference with ATP production by respiration. Although mitochondria are considered to be the main target site of metformin, we also observed that the *hd*PCA signal was reduced for proteins involved in one-carbon and nucleotide (deoxynucleoside triphosphate [dNTP]) metabolism, which is in line with previous observations (Figure 4D; Cabreiro et al., 2013, Janzer et al., 2014).

Nucleotide synthesis requires methyl groups from folate intermediates produced in one-carbon metabolism. We observed that dNTP levels were reduced in yeast grown in metformin (Figure 4E). Overall, *hd*PCA reporter signals for catabolic processes increased, but those of anabolic processes were reduced in cells grown in metformin (Figure 3).

### *hd*PCA Predicts Genes Implicated in Cancers and Chronological Aging

Proteins that stimulate chronological aging showed a reduced *hd*PCA signal on metformin, consistent with observations of increased yeast longevity (Figure 4F; Borklu-Yucel et al., 2015). Metformin increased median survival time by 16 days, a 3.2-fold increase over the control (Figure 4G). The *hd*PCA hits involved in chronological cell aging included Dnm1, a protein required for mitochondrial fission, a process inhibited by metformin in diabetic mice (Wang et al., 2017), and three TOR pathway proteins (Sch9, Gcn2, and Cpa2). The TOR pathway components were also enriched among GO biological processes showing a reduced *hd*PCA signal in metformin (Figure S1B). Metformin inhibits the TOR pathway in breast cancer cells (Dowling et al., 2007), and in *C. elegans*, it inhibits TORC1 through suppression of mitochondrial respiration, linking energy metabolism with the TOR signaling pathway (Dowling et al., 2007, Wu et al., 2016). The homolog of the TOR substrate ribosomal S6 kinase, Sch9, was the most significant *hd*PCA hit (p = 2.04 × 10^−6^), and S6 kinase is inhibited by metformin (Dowling et al., 2007). Finally, the *hd*PCA data suggest that metformin induces a DNA repair response in yeast, of particular interest for alternative applications of metformin for cancer prevention and therapy. *SWI/SNF* and *INO80* chromatin remodeling complexes in metformin-mediated DNA repair mechanism components were prominently enriched in the *hd*PCA data (Figure S1C).

### Metformin Provokes a Cellular State that Resembles Iron Deficiency

**Figure 5:**
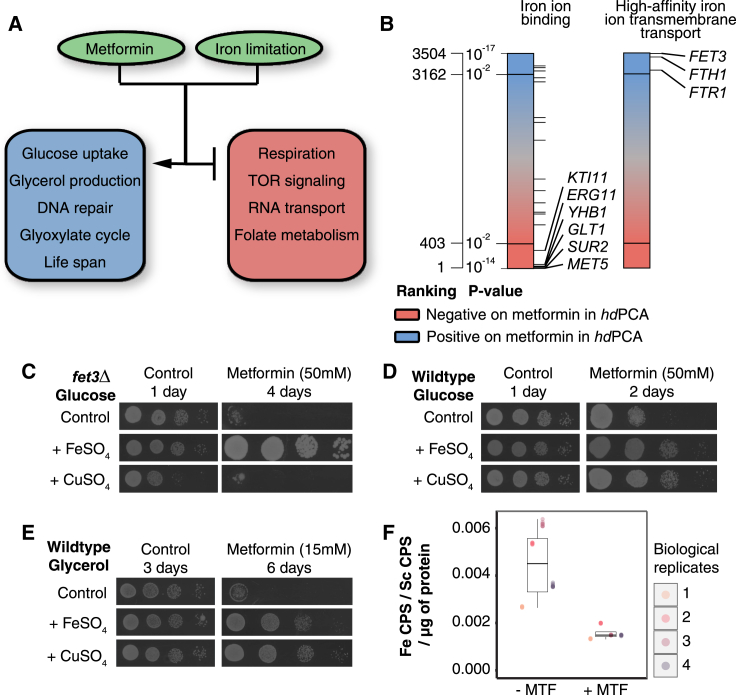
Metformin Treatment Interferes with Iron Homeostasis (A) Processes affected by metformin treatment according to *hd*PCA data are also modulated by iron limitation. (B) Metformin *hd*PCA data on proteins involved in high-affinity iron ion uptake and proteins binding iron. (C) Growth of *fet3Δ* in the presence of metformin and CuSO_4_ or FeSO_4_. (D) Growth of wild-type yeast in the presence of metformin and CuSO_4_ or FeSO_4_. (E) Growth of wild-type yeast under respiratory conditions with metformin and CuSO_4_ or FeSO_4_. (F) Protein was extracted from yeast cultures incubated in the presence (+ MTF) or absence (− MTF) of metformin. The concentration of ^56^Fe bound to the protein was determined by inductively coupled plasma mass spectrometry (ICP-MS) (8 technical replicates are shown of 4 biological replicates for each condition). CPS, counts per second; Sc, scandium. See also Figure S2 and Table S8.

We found that iron deficiency was a common theme among cellular processes affected by metformin. A reduction in respiratory capacity, increased glucose uptake, increased glycerol production, inhibition of the TOR pathway, increased lifespan, and activation of DNA repair are all found under conditions of iron limitation in yeast and/or other organisms (Figure 5A; Ansell and Adler, 1999, Dimmer et al., 2002, Dongiovanni et al., 2008, Ohyashiki et al., 2009, Schiavi et al., 2015, Szüts and Krude, 2004). Furthermore, other cellular functions found in the *hd*PCA data, such as RNA transport, vesicle-mediated transport, the glyoxylate cycle, and folate metabolism, have been linked to iron deficiency (Beier et al., 2015, Jo et al., 2009, Oppenheim et al., 2000, Zhang et al., 2014). In addition, iron-binding proteins showed a reduced, and proteins that counteract iron depletion an increased, *hd*PCA signal (Figure 5B; Jo et al., 2009).

**Figure S2:**
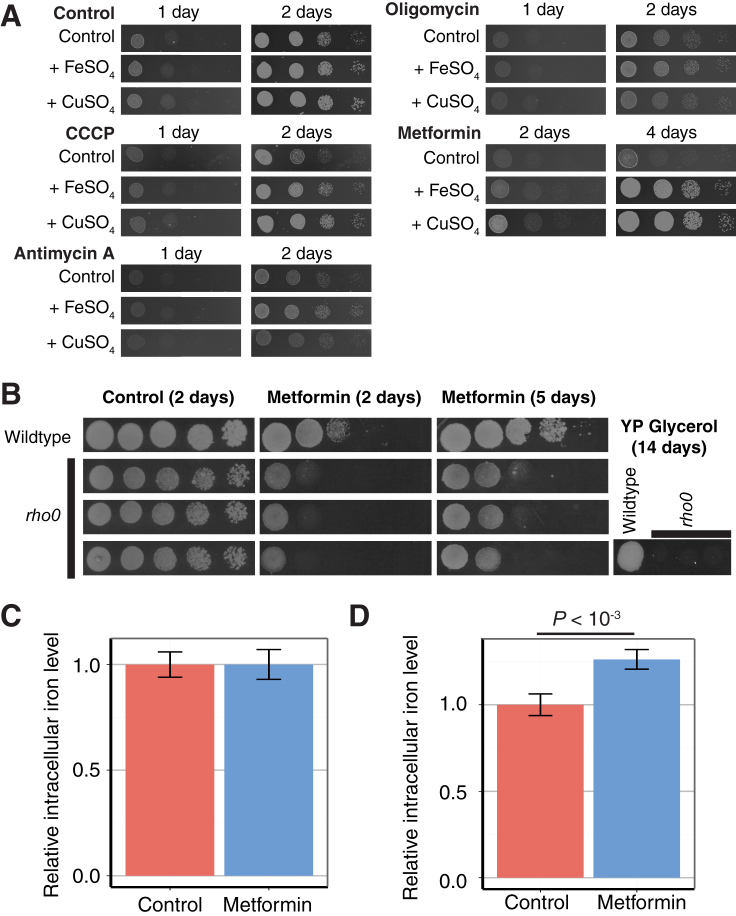
Metformin More Closely Relates to Iron Homeostasis than Respiration Despite the Lack of an Effect on Intracellular Iron Levels, Related to Figure 5 (A) Growth of wild-type yeast on YP raffinose (2%) in the presence of different inhibitors of oxidative phosphorylation (CCCP at 20 μM, antimycin A at 0.1 μg/ml, oligomycin at 3 μg/ml) and in the presence or absence of additional FeSO_4_ (500 μM) or CuSO_4_ (500 μM). Raffinose is a carbon source that is consumed through both fermentation and respiration. (B) Growth of wild-type yeast and three independent respiration-deficient (‘petite’) *rho0* strains in the presence or absence of metformin (50 mM) on YPD. Respiration-deficient strains do not grow in respiratory conditions (YP glycerol). (C) Intracellular iron content of yeast cultures in exponential growth phase (O.D._600_ = 1) after treatment with metformin, relative to the control condition. Error bars indicate standard deviation (n = 10). (D) Intracellular iron content of yeast cultures in stationary growth phase after treatment with metformin, relative to the control condition. Error bars indicate standard deviation (n = 3).

An increase in *hd*PCA signal for the high-affinity iron uptake system provides a more direct link between the *hd*PCA data and iron deficiency (Figure 5B). Yeasts were more sensitive to metformin in deletion strains of the ferro-O_2_-oxidoreductase *FET3,* which is directly involved in copper-dependent high-affinity iron uptake (Figure 5C). Iron supplementation overcame the growth deficiency of *fet3Δ* in the presence of metformin. Furthermore, addition of iron (FeSO_4_) or copper (CuSO_4_) to growth medium made wild-type yeast less sensitive to the drug (Figure 5D). The antagonistic effect of copper on metformin-mediated growth inhibition required Fet3, which suggests that copper mainly improves yeast growth by stimulation of copper-dependent iron uptake. Under respiratory growth conditions, which require more iron than fermentable conditions, the supplementation of iron (or copper) countered the effects of metformin on growth more profoundly (Figure 5E). This clear growth interaction between iron or copper and metformin was not found for three electron transport inhibitors (carbonyl cyanide *m*-chlorophenyl hydrazone [CCCP], antimycin A, and oligomycin) (Figure S2A). Together with the observation that respiration-deficient yeast remained sensitive to metformin (Figure S2B), these results point to a relationship between iron and copper and metformin, independent of oxidative phosphorylation. Overall, these results suggest that metformin either interferes with iron uptake or with intracellular iron homeostasis. We found that metformin did not reduce the intracellular iron content during exponential growth (Figure S2C) and even increased intracellular iron levels in stationary phase cultures (Figure S2D). However, the global iron deficiency-like response seems to originate from a redistribution of iron inside the cell. The level of protein-bound iron is reduced upon metformin treatment (Figure 5F; Student’s t test, p < 0.05). This result indicates that interference with iron homeostasis may lie at the heart of how metformin influences a whole spectrum of cellular processes. Further research in human cells or in the human microflora should establish whether this relationship between iron and metformin is preserved and relevant to its therapeutic actions.

## Discussion

Our observations suggest that metformin interferes with the distribution of iron in the cell. Iron supplementation partially compensates for this perturbation. Indeed, iron metabolism is intrinsically linked to changes in central glucose metabolism, the etiological cause of diabetes. Glycolysis itself most likely started as a Fe(II)-catalyzed network of sugar phosphate interconversions in early evolution, and a unifying function of mitochondria in all eukaryotes is the assembly of FeS clusters, a process intrinsically linked to iron transport, whereas the tricarboxylic acid (TCA) cycle and electron transport also use the FeS clusters in its catalysis (Keller et al., 2014, Lill et al., 2014). Interestingly, it was recently shown that interference with these core mitochondrial processes could affect other pathways, including TOR signaling (Wu et al., 2016). Our results, linking metformin directly to intracellular iron transport, may bear directly on observations in humans. Iron excess caused by hereditary factors, nutrition, or medical intervention is a diabetes risk factor (reviewed in Simcox and McClain, 2013). A low-iron diet or iron chelation therapy improves glycemia in diabetes-prone, leptin-deficient mice (Cooksey et al., 2010). Metformin treatment leads to a trend of reduction in serum iron levels (Luque-Ramírez et al., 2007). In cells, iron levels remain intrinsically linked to metabolic activity, both directly over enzymes that use ferrous iron or FeS clusters for their catalytic function and indirectly over their involvement in Fenton chemistry, being a major contributor to cellular redox metabolism by catalyzing the formation of superoxide from hydrogen peroxide. Interfering with iron metabolism will certainly re-configure metabolism on the cellular scale, and because metabolic reconfigurations globally affect the cellular response to all sorts of perturbations on the transcriptome, proteome, and metabolome (Alam et al., 2016), major physiological responses can be explained without the need of the drug hitting a single specific target. Our results warrant a closer investigation of the effects of metformin on iron homeostasis, accessibility, and distribution at possible sites of metformin activity, including the gut and the liver.

With *hd*PCA, we were able to uncover the effect of metformin on a broad set of cellular processes, and we found cross-species conservation of core mechanisms of metformin treatment in yeast. This opens up the possibility for future research on metformin with this model organism. Furthermore, the overlap between the *hd*PCA data and GWA studies related to diabetes and prostate cancer may suggest that the homologs should be considered further as predictors of disease.

As a broader application, we can prescribe a general approach to use *hd*PCA to map out specific biochemical pathways and networks. For instance, if we did not know anything about the TOR pathway, the fact that we observe a reduction in *hd*PCA signal for Tor1 could serve as a launching point to identify other pathway components. This next step is achieved by performing a heteromeric PCA screen with Tor1 as bait and our existing arrays of most other proteins as prey (Tarassov et al., 2008). We previously demonstrated that the entire canonical TOR pathway could be mapped in this way, and we also discovered a novel pathway linking Tor to modulation of the arginine methlytransferase activity of Hmt1 in yeast and resultant control of cell cycle progression (Messier et al., 2013, Remy and Michnick, 2001).

Finally, *hd*PCA provides a simple strategy to globally profile cellular responses to external perturbations that can be applied widely to study the mechanisms of action of bioactive molecules and their effects on intended target or unsuspected off-target effects. Identifying off-target effects is particularly important for determining both potential liabilities and added benefits of molecules (Caskey, 2007). Applications of *hd*PCA include the general study of the effect of environmental stresses, revisions of mechanisms of action of commercialized drugs, comparison of off-target effects within a drug family, and elucidation of the harmful effects of toxins.

## STAR★Methods

### Key Resources Table

**Table:** 

REAGENT or RESOURCE	SOURCE	IDENTIFIER
**Chemicals, Peptides, and Recombinant Proteins**		

Bathophenantrolinedisulfonic acid disodium salt hydrate (BPS)	Sigma-Aldrich	Cat#146617; CAS 52746-49-3
Hygromycin B	Wisent Bioproducts	Cat#450-141-XL; CAS 31282-04-9
Metformin-HCl	Jean Coutu	CAS 1115-70-4
Methotrexate	BioShop Canada	Cat#MTX440; CAS 59-05-2
Nourseothricin-dihydrogen sulfate	Werner Bioagents	Cat#5.005.000; CAS 96736-11-7
Rapamycin	BioShop Canada	Cat#RAP004; CAS 53123-88-9
Ethidium bromide	BioShop Canada	Cat#ETB444.50; CAS 1239-45-8
Carbonyl cyanide m-chlorophenylhydrazone (CCCP)	Sigma-Aldrich	Cat#C2759-250MG; CAS 555-60-2
Antimycin A	Sigma-Aldrich	Cat#A8674-25MG; CAS 1397-94-0
Oligomycin	Sigma-Aldrich	Cat#495455-10MG; CAS 1404-19-9
Acetonitrile	Greyhound	Cat#Bio-012041; CAS 75-05-8
Water ULC MS	Greyhound	Cat#Bio-23214102; CAS 7732-18-5
Formic acid	Fluka	Cat#O6454; CAS 64-18-6
Ammonium acetate	Greyhound	Cat#Bio-012441; CAS 631-61-8
Acetic acid	Fluka	Cat#49199-50ml; CAS 64-19-7
Ammonium hydroxide	Sigma-Aldrich	Cat#221228-100ml; CAS 1336-21-6
dATP/dGTP/dTTP/dCTP	Bioline	Cat#39025
Internal standard mix for ICP-MS	Agilent	Cat#5188-6525
Multi-element calibration standard-2A	Agilent	Cat#8500-6940

**Experimental Models: Organisms/Strains**		

*S. cerevisiae*: BY4741	Brachmann et al., 1998	ATCC 201388
*S. cerevisiae*: mDHFR PCA strain collection	Tarassov et al., 2008	N/A
*S. cerevisiae*: deletion strain collection	Giaever et al., 2002	N/A

**Recombinant DNA**		

Plasmid: pHLUM	Mülleder et al., 2012	N/A
Plasmid: pYes2-P_ACT1_-pHluorin	Orij et al., 2009	N/A
Plasmid: pYes2-P_ACT1_-mtpHluorin	Orij et al., 2009	N/A

**Software and Algorithms**		

Fiji (v1.45b)	Schindelin et al., 2012	http://imagej.net/Fiji/Downloads; RRID: SCR_002285
R (v3.2.3)	The R project	https://www.r-project.org/; RRID: SCR_001905
Cytoscape	Smoot et al., 2011	http://cytoscape.org; RRID: SCR_003032
limma (R)	Bolstad et al., 2003, Ritchie et al., 2015	http://bioconductor.org/packages/release/bioc/html/limma.html; RRID: SCR_010943
affy (R)	Bolstad et al., 2003, Gautier et al., 2004	http://bioconductor.org/packages/release/bioc/html/affy.html; RRID: SCR_012835
cyber-T (R)	Baldi and Long, 2001, Kayala and Baldi, 2012	N/A
GOstats (R)	Falcon and Gentleman, 2007	http://bioconductor.org/packages/release/bioc/html/GOstats.html
biomaRt (R)	Durinck et al., 2009	http://bioconductor.org/packages/release/bioc/html/biomaRt.html
EnrichmentMap (Cytoscape)	Merico et al., 2010	http://apps.cytoscape.org/apps/enrichmentmap
AutoAnnotate (Cytoscape)	Kucera et al., 2016	http://apps.cytoscape.org/apps/autoannotate
MassHunter	Agilent	https://www.agilent.com/en/products/software-informatics/masshunter-suite/masshunter/masshunter-software; RRID: SCR_015040

### Contact for Reagent and Resource Sharing

Further information and requests for resources and reagents should be directed to and will be fulfilled by the Lead Contact, Stephen Michnick (stephen.michnick@umontreal.ca).

### Experimental Model and Subject Details

#### *Saccharomyces cerevisiae* strains

DHFR PCA strains have open reading frames of proteins of interest tagged with DHFR fragments [1,2] and [3] by homologous recombination in *Saccharomyces cerevisiae* BY4741 (*MAT*a *his3Δ1 leu2Δ0 met15Δ0 ura3Δ0*) and BY4742 (*MAT*α *his3Δ1 leu2Δ0 lys2Δ0 ura3Δ0*) strains (Brachmann et al., 1998) respectively, as described in Tarassov et al. (2008). BY4741 was used in the chronological lifespan assay and the metal, electron transport inhibitor and carbon source spot assays. BY4743 and independent petite, respiration-deficient (*rho0*) versions of diploid strain BY4743 were used for the spot assay focused on respiration deficiency. For mass spectrometry analysis, BY4741 was transformed with pHLUM to make the strain prototrophic for histidine, leucine, uracil and methionine (Mülleder et al., 2012). For intracellular pH measurements, BY4741 was transformed with either pYes2-P_ACT1_-pHluorin or pYes2-P_ACT1_-mtpHluorin (Orij et al., 2009). Deletion strains, including *fet3Δ*, originate from BY4741 with a *kanMX* cassette at the position of the deleted gene (Giaever et al., 2002).

#### *Saccharomyces cerevisiae* growth conditions

Growth media were based on yeast extract-peptone-dextrose (YPD; 20 g/L bactopeptone,10 g/L yeast extract, 2 (w/v) % glucose) or synthetic complete (SC; 1.74 g/L yeast nitrogen base, 5 g/L ammonium sulfate, 1x amino acid mix, 2% glucose) formulations. For solid medium, 20 g bacto-agar per liter was added or 40 g/L Noble agar in the case of the *hd*PCA screen. The 1x amino acid mix consists of 0.04 g adenine sulfate, 0.02 g uracil, 0.04 g tryptophan, 0.02 g histidine-HCl, 0.02 g arginine-HCl, 0.02 g methionine, 0.03 g tyrosine, 0.06 g leucine, 0.03 g lysine-HCl, 0.05 g phenylalanine, 0.1 g glutamic acid, 0.1 g aspartic acid, 0.15 g valine, 0.2 g threonine and 0.375 g serine per liter. Dropout media was prepared by leaving out the appropriate compound from the amino acid mix. YPD medium has a pH of 7.1 while the pH of unbuffered SC medium is 4.8. After discovery that metformin sensitivity increases with pH, the pH of SC medium was increased to 7 in further experiments. Ammonium sulfate reduces metformin sensitivity. Details on pH, ammonium sulfate presence and dropout conditions are given below for each experiment. Methotrexate was purchased from BioShop Canada (Montréal, Canada), nourseothricin (ClonNAT) from Werner Bioagents (Jena, Germany), hygromycin B was from Wisent Bioproducts (Saint-Jean-Baptiste, Canada), rapamycin from BioShop Canada (Montréal, Canada) and metformin-HCl from Jean Coutu (Montréal, Canada).

### Methods Details

#### Homomer dynamics PCA (*hd*PCA)

The DHFR PCA procedures have been previously described (Tarassov et al., 2008). Briefly, first, 3,504 *MAT*a strains harboring *ORF.X-DHFR-F[1,2]-nat1* fusions were arrayed from 96-well glycerol stocks in 384-format on YPD agar plates (Omnitrays, Nunc) containing 100 μg/mL nourseothricin using a robotically manipulated 96-pin tool (0.787 mm flat round-shaped pins, custom AFIX96FP3 BMP Multimek FP3N, V&P Scientific, San Diego, CA). The same process was repeated for 3,504 *MAT*α strains with *ORF.X-DHFR-F[3]-hph* fusions but on YPD with 250 μg/mL hygromycin B instead of nourseothricin. The strains, now on a total of 2 × 15 plates, were grown for 24 hours at 30°C. Next, the strains were condensed into a 1,536-format using a robotically manipulated 384-pin tool (0.457 mm flat round-shaped pins, custom AFIX384FP1 BMP Multimek FP1N, V&P Scientific, San Diego, CA) on YPD agar plates with 100 μg/mL nourseothricin for *MAT*a strains and 250 μg/mL hygromycin B for *MAT*α strains. The strains, now on a total of 2 × 4 plates, were grown for 24 hours at 30°C. For the mating step, the *MAT*a and *MAT*α strains were combined one-to-one so that each resulting diploid strain had one gene tagged on both alleles with one of the DHFR fragments. The strains were combined on YPD agar plates using a robotically manipulated 1,536-pin tool (0.457 mm flat roundshaped pins, custom AFIX1536FP1 BMP Multimek FP1N, V&P Scientific, San Diego, CA). The strains, now on four plates, were grown for 24 hours at 30°C. For the diploid selection step, the strains were transferred to synthetic complete agar medium without lysine and without methionine and with 100 μg/mL nourseothricin and 250 μg/mL hygromycin B and were incubated for 48 h at 30°C. This step was repeated to further select for diploid cells. Finally, the strains were printed on SC agar medium without adenine (pH 4.8) containing 4% (w/v) Noble agar, 2% (w/v) glucose, 1.74 g/L YNB without ammonium sulfate, and methotrexate (200 μg/mL) to select for DHFR reconstitution in the presence or absence of 50 mM metformin (from 1 M stock in water) or 2 nM rapamycin (from 2 mM stock in ethanol). Pin tips were wetted in 75% glycerol solution before printing on methotrexate selection plates in order to dissolve colonies and obtain a small amount and a homogeneous colony material on all the pins. Four replicate plates were used giving a final number of 16 plates for each condition. Printed methotrexate plates were incubated at 30°C throughout the imaging process and individual plates were photographed on days 1 to 5, 7, 10, 12 and 14 with a 4-megapixel Canon digital camera (Powershot A520).

#### Induction of respiration deficiency with ethidium bromide

Diploid strain BY4743 was grown in SC medium until exponential phase, at which point ethidium bromide (10 μg/ml; BioShop) was added and incubated at 30°C, shaking (Goldring et al., 1970). After 2h, samples were diluted in PBS (0.15 M NaCl, 0.02 M K_2_HPO4, pH 7.6) and plated on YPD. Independent petite colonies were tested for absence of growth on YP glycerol (3%) to confirm respiration deficiency.

#### Carbon source, metal ion, electron transport chain inhibitor and respiration-deficiency spot assays

BY4741 cells were spotted on YPD agar plates in 10-fold dilutions in the presence or absence of metformin (50 mM). For the carbon source experiments, 2% glucose was replaced by 0.5% or 4% glucose, 2% galactose, 2% raffinose or 3% glycerol. For the metal ion spot assay, 500 μM FeSO_4_ or 500 μM CuSO_4_ were added to the medium with glucose (2%) or glycerol (3%) as carbon source. In the case of glucose, both BY4741 and *fet3Δ* were tested. For the spot assay with electron transport chain inhibitors, CCCP (final concentration: 20 μM), antimycin A (0.1 μg/ml), oligomycin (3 μg/ml) or metformin (15 mM) were added to YP medium with 2% raffinose, with addition of 500 μM FeSO_4_ or 500 μM CuSO_4_.

#### Cytoplasmic and mitochondrial pH

The pH marker protein pHluorin, of which the fluorescence emission spectrum depends on surrounding pH, was used to measure cytoplasmic and mitochondrial pH. Strain BY4741 was transformed with pYes2-P_ACT1_-pHluorin for cytoplasmic pH measurements or pYes2-P_ACT1_-mtpHluorin for mitochondrial pH measurements. The protocol was followed as described by Orij et al. (Orij et al., 2009). For calibration, pHluorin-positive and –negative strains were grown to an O.D._600_ of 1 in SC medium (without uracil for pHluorin-positive strains) and after centrifugation for 5 min at 4,000 rpm, cells were resuspended in PBS containing 100 μg/ml digitonin. After 10 min, cells were washed with PBS and put on ice. The cells were then precipitated at 4,000 rpm for 5 min and transferred to citric acid/Na_2_HPO_4_ buffer at pH values from 5.0 to 8.7, in black polystyrene clear-bottom 96-well plates and at a final cell density of O.D._600_ 0.5. A SpectraMax Gemini-XS spectrofluorometer (Molecular Devices) was used to measure fluorescence emission at 512 nm after excitation at 390 and 470 nm. Fluorescence from a pHluorin-negative wild-type culture was subtracted from the measurements. The ratio of emission intensities from excitation at 390 and 470 nm was plotted against the corresponding buffer pH.

To study the influence of metformin on the mitochondrial and cytoplasmic pH, cultures of wild-type BY4741, BY4741 with pYes2-P_ACT1_-pHluorin and BY4741 with pYes2-P_ACT1_-mtpHluorin were grown in SC medium (pH7) with ammonium sulfate and in the presence or absence of metformin (50 mM) and with (wild-type) or without uracil (pHluorin-positive strains), at 30°C with regular shaking within the SpectraMax Gemini-XS spectrofluorometer and at starting O.D._600_ of 0.2. Absorbance at 600 nm (cell density) and emission at 512 nm after excitation at 390 and 470 nm was determined for at least 5 technical replicates at 6 time points over the course of 11 h. The ratio of emission at 390 and 470 nm was calculated after subtraction of the values with background fluorescence from the wild-type strain and correction for cell densities. Results were confirmed in a repetition of the assay.

#### Chronological lifespan assay

BY4741 was incubated in SC medium as described in Matecic et al. (Matecic et al., 2010) in 3 mL overnight, reincubated in 50 mL for 24h and finally in 500 mL for another 24h, always shaking at 30°C. Next, the culture was split into 4 mL cultures, for each condition three replicates with metformin (50 mM) and three with water (mock), and further incubated at 30°C. Cell survival was tracked by plating out individual cells and counting the number of colony-forming units. Cell survival was quantified by determination of the time point at which 50% of the culture had died, with 100% survival taken at the day the cultures reached their maximum concentration of living cells (day 5). The extent to which metformin prolongs lifespan was taken as the ratio of the median survival time in the presence of metformin over the median survival in the control condition. The results were confirmed in a repetition of the assay.

#### Analysis of dNTP content by LC-MS/MS

At mid-log phase, 10 O.D. units of BY4741 (with pHLUM) grown in liquid SC (pH 7; without ammonium sulfate, uracil or adenine; with 1 g/ml glutamate as main nitrogen source) medium in the absence or presence of metformin (5 mM and 50 mM) were quenched by rapid cold methanol (de Koning and van Dam, 1992). Cell pellets were resuspended in 200 μl of organic extraction buffer (75:25 acetonitrile (Bio-012041): water (Bio-23214102), 0.2% formic acid (Fluka O6454) and lysed mechanically by acid wash glass beads using a FastPrep-24 (MP Biomedicals). A second extraction was performed with 200 μl of water. Supernatants from both extraction steps were combined, evaporated in a SpeedVac concentrator and resuspended in 100 μl of 10 mM ammonium acetate (Greyhound 012441) in 7:3 water:acetonitrile pH 6, and subsequently quantified by LC-MS/MS as described by Hofmann et al. (2012). Briefly, nucleotides were separated by ion-exchange HPLC (Agilent 1290 Infinity with autosampler tray cooled at 4°C) using a weak anion exchange column (Biobasic AX 2.1 id x 50 mm column, Thermo Scientific 73105-052130). This was achieved using 10 mM ammonium acetate in water:acetonitrile 7:3 at pH 6 for mobile phase A and 1 mM ammonium acetate (Greyhound 012441) in water:acetonitrile 7:3 pH 10.5 for mobile phase B at 30°C. The pH was adjusted with acetic acid or ammonium hydroxide. The gradient started at 0% B, was increased to 35% B from 1 to 2.5 min, further increased to 65% B from 5 to 7 min, and then increased to 100% B from 10 to 10.5 min, which was held until 15 min. Post-run equilibration was done using 100% A for 5 min. The flow rate was 0.15 mL/min for the first minute and then 0.25 mL/min until 15 min. The volume injected was 5 μl. The ionization mode of the mass spectrometer was positive ESI. The MRM transitions are summarized in the following table:

#### SRM setup

**Table:** 

	Precursor ion	Produced ion	Dwell	Fragmentator (V)	Collision energy (V)
dATP	492	136.1	100	128	21
dCTP	468	112.1	100	74	13
dGTP	508	152	100	78	13
dTTP	483	81	100	128	9
ATP	508.1	136	25	128	33
ADP	428.2	136	25	128	21
AMP	348.2	136	25	112	13

A standard curve was prepared using 10 μM of dATP (Bioline 39025), dCTP (Bioline 39025), dGTP (Bioline 39025) and dTTP (Bioline 39025) and making threefold dilutions (1:3:3:3:3:3:3). Analysis was done using the MassHunter software (Agilent).

#### Intracellular iron concentration

Wild-type yeast (BY4741) was incubated in SC medium without ammonium sulfate (pH 6.5) until stationary phase (36h) and cells were processed according to the protocol of Tamarit et al. (2006). Cells (20 μl) were collected by centrifugation for 5 min at 3,000 rpm, resuspended in 500 μl 3% nitric acid and left for 16h at 95°C in tightly closed tubes. Next, cell debris was precipitated by centrifugation for 5 min at 5,000 rpm and 400 μl of supernatans was mixed with 160 μl of 38 mg/ml sodium ascorbate, 320 μl of 1.7 mg/ml BPS and 126 μl ammonium acetate (3x dilution from a saturated solution). After 5 min, the absorbance of the iron-chelator complex was measured at 535 nm with a LS-50B spectrometer (Perkin-Elmer). The mean absorbance value at 535 nm of three blank (no cells) samples and absorbance levels at 680 nm (for each sample separately) were subtracted from the 535 nm results. Five technical replicates were used in the experiment. The results were confirmed in a repetition of the assay.

#### Analysis of concentration of protein-bound iron by ICP-MS

The TAP-tagged LEU1-TAP BY4741 strain (Dharmacon-Horizon Discovery, Cambridge, UK) was grown in 50 mM metformin containing YPD or plain YPD broth until the culture reached an O.D._600_ nm of 1.0 (4 replicates for each condition). The yeast cells were then harvested by centrifugation, washed and lysed by beadbeating and vortexing 25 times for one minute in lysis buffer (150 mM of NaCl, 50 mM of Tris-HCl pH 8.0, 5 mM of EDTA, 10% (v/v) glycerol, 0.2% (v/v) Nonidet P-40, 1 mM of NaF, 1 mM phenylmethylsulfonyl fluoride (PMSF) and Complete mini EDTA-free protease inhibitor cocktail from Roche, Mannheim, Germany; Kaiser et al., 2008). The lysed yeast cell supernatants for both conditions were clarified by centrifugation at 21,000 g for 30 min, and the protein concentrations were determined by a Bradford assay (Bradford, 1976). The resulting protein extract sample was diluted sevenfold in lysis buffer and small molecules were removed from that suspension by a 14,000 g centrifugation at 4°C during 30 min through an Amicon Ultra 0.5 μL MWCO 3000-Da microcolumn (Millipore-Sigma, St-Louis USA). The protein extract sample that remained above the Amicon membrane was flash-frozen and lyophilized. The strain LEU1-TAP was chosen for this experiment because in parallel, Leu1 was purified for determination of the concentration of iron bound to Leu1. Although a reduction in iron bound to Leu1 was observed upon metformin administration, this difference was not statistically significant (data not shown).

The protein extract sample was digested by adding 22.9 μl of concentrated HNO_3_ (70%) and incubating each sample for 4 hours at 70°C. After digestion, 796 μl of de-ionized HPLC-grade water and 4 μl of an 10 μg/ml of internal standard solution (Agilent 5188-6525) was added to each sample such that the final concentration of HNO3 was 2% and that of each element in the internal standard mixture was 50 ng/ml.

A set of calibration standards was made by diluting the concentrated calibration standard mixture (Agilent 8500-6940). The calibration curve and the samples were then injected into an Agilent ICP-MS 7900 using a microflow nebulizer (MicroMist; Cat No.: G3266-80004) and the SPS4 autosampler (Model No.: G8410A). The measurements were obtained in two blocks (calibration standards and samples) and the sequence of measurements within each block was randomized. Calibration blanks (2% HNO_3_) were run after every 9 samples. The acquisition settings we used were: Integration time of 0.06 s for Scandium [45] (the internal standard) and 0.399 s for Iron [56], Stabilization Time of 5 s, 8 internal ICP-MS measurement replicates with 200 sweeps per replicate. The peristaltic pump settings were: sample uptake for 30 s and stabilization time of 20 s.

The calibration curve was used to determine the limit of detection, ensure that our measurements lie in the linear range of the ICP-MS and in the “Pulse Mode.” We report the unprocessed counts per second values. The counts per second (CPS) of Fe in each sample were normalized by CPS of Sc and then divided by the amount of protein in the sample. The raw and processed data can be found in Table S8.

#### Deletion miniscreen

Deletion strains (Giaever et al., 2002) of the 96 proteins with the most significant metformin-included changes in *hd*PCA signal were tested for growth on SC agar plates (pH 7) without ammonium sulfate and in the presence or absence of metformin (50 mM). Unavailable deletion strains were replaced by deletion strains of proteins next on the list of significant *hd*PCA hits. A set of 48 negative control strains were taken from proteins without significant change in *hd*PCA signal. Growth of the strains, as 10-fold dilutions in a spot assay, was monitored over 5 days. Reduction or increase in sensitivity to metformin was determined by comparison of the growth of each strain to the general trend of growth from strains with similar fitness on the control plates.

### Quantification and Statistical Analysis

#### Data analyses of the *hd*PCA

##### Quantification of colony growth

Resulting images were analyzed using imaging software Fiji (v1.45b) (Schindelin et al., 2012). To assess colony growth, a custom-made macro was created to measure the integrated density of each colony. First, the position of the top left colony in the grayscale image was determined together with the angle of the colony rows relative to the image frame. These coordinates were used for an initial estimation of the colony positions. For each colony, a 17x17 pixel area was screened around the estimated central position to find the central gravity point in pixel intensity. From this central pixel, the first pixels with background intensity in the left, right, bottom and up directions were determined to get a new central pixel. This process was repeated to get the final central pixel and the borders of the colony in the four directions. An oval was created and analyzed for integrated density, which is the mean density times the area.

##### Data normalization and identification of hdPCA significant hits

All statistical analyses were performed using a custom-made pipeline in R (v3.2.3), unless mentioned otherwise. The integrated densities calculated in the previous section were log_2_ transformed. Subsequently, they were normalized by two methods. First, the quantile normalization from the Bioconductor limma package was used to correct for technical plate-to-plate variabilities (Bolstad et al., 2003, Ritchie et al., 2015). It was applied on colonies of each replicate within a given condition, in order to make their logged densities have the same distribution. Second, the cyclic locally weighted scatterplot smoothing (LOESS) normalization from the Bioconductor affy package was used to correct for the effect of rapamycin and metformin on colony growth (Bolstad et al., 2003, Gautier et al., 2004). The local regression principle of LOESS normalization takes into account the colony size, which affects the basal effect of the drugs on growth. For instance, colonies with slow growth rate were affected by metformin more than those with high growth rate. This gives rise to a variable factor affecting the different colonies. The cyclic LOESS was applied on colonies belonging to each pair of plates in order to calculate the variable effect, which was then subtracted from the logged density of these colonies. The variable effect was assessed using a local regression to fit a curve in a set of colonies plotted in function of their logged densities in a plate pair. For this analysis, optimal results were obtained by setting the normalize.loess function parameters as follows: normalize.loess(data, epsilon = 1, log.it = F, span = 0.05, maxit = 5). Finally, strains growing better on a drug versus control and on control versus a drug were identified using a regularized t test that was implemented in the Cyber-T software R script (Baldi and Long, 2001, Kayala and Baldi, 2012). When the number of replicates within a condition is small, the regularized t test is preferred over the traditional Student’s t test, as it estimates a regularized variance of the normalized densities associated with each strain under each condition. Using a Bayesian probabilistic approach, the regularized variance of a strain is robustly estimated as a weighted average of the variances of this strain and of its neighbors. These neighbors are defined by a spanning window, after ranking strains by their normalized density averages. For our analysis, the Cyber-t test was performed with the default settings, except for the adjusted degrees of freedom parameter, called conf, that was set to 15, giving thus the neighbors more importance in estimating the regularized variance. The Cyber-t test was separately applied on strains of each day, generated *P-*values were then adjusted by the method of Benjamini and Hochberg for multiple testing (Benjamini and Hochberg, 1995). Subsequently, strains of the different days were combined and only those having a *P-*value < 0.01 in at least two days were selected to form the set of hits of each test (control versus a drug and a drug versus control).

#### Comparison of rapamycin *hd*PCA data with rapamycin data of external studies

Data of rapamycin studies that involve deletion strain libraries (Dudley et al., 2005, Kapitzky et al., 2010, Parsons et al., 2004, Xie et al., 2005), mRNA expression analysis (Düvel et al., 2003, González et al., 2013, Hardwick et al., 1999, Huang et al., 2004), mass spectrometry proteomics (protein abundance) (Dephoure and Gygi, 2012, Iesmantavicius et al., 2014, Paulo and Gygi, 2015), a GFP library (protein abundance and localization changes) (Chong et al., 2015) and mass spectrometry phosphoproteomics (Huber et al., 2009, Iesmantavicius et al., 2014, Paulo and Gygi, 2015, Soulard et al., 2010) were collected and merged into five datasets according to their study categories: mRNA abundance, protein abundance, phosphorylation levels, deletion strain fitness and flux (change in protein localization). Quantitative data were used whenever available. Rapamycin fitness data from deletion screens was converted into numeric values with 1 for increased resistance, −1 for increased sensitivity and 0 for no effect. The individual deletion screen datasets were merged by simple addition of the values of each gene across the datasets to obtain a compiled deletion fitness dataset. For mRNA expression analysis, the mean change in expression of the four datasets was taken. For the GFP protein abundance dataset, the difference in abundance between the control condition and rapamycin was calculated as the mean difference from four time points between 300 and 540 min of rapamycin exposure. The resulting dataset was merged with mass spectrometry proteomics abundance data after normalization by dividing each set individually by its standard deviation. Flux data were collected by taking the top 100 proteins, based on Z-score, moving away or toward 16 subcellular compartments at two time points (380 and 460 min after rapamycin exposure). For each protein, the total movement was taken as the sum of all Z-scores (in absolute values). Proteins absent in the 32 top 100 lists but present in the GFP library were given a Z-score value of 0. For phosphoproteomics data compilation, the qualitative dataset from Soulard et al. (2010) was incorporated into the merged set as numeric values (−1 for reduced phosphorylation and 1 for increased phosphorylation) while the four quantitative datasets were added to the compiled data after normalization of each set individually through division by the standard deviation. The rapamycin *hd*PCA data were ranked according to *P*-value. Ranking by *P-*value instead of colony size difference reduces the influence of noise typically generated from measurements of smaller colonies. The significance of overlap between two compiled datasets was calculated with the hypergeometric test with a universe that consisted of genes/proteins tested in both experiments and the lowest *P*-value was retained among comparisons that included the top 1, 5, 10, 15, 20 and 25% of both ranked datasets.

To determine the *P*-value threshold at which *hd*PCA data significantly overlaps with other datasets, the overlap between the top 20% results of external datasets was compared to ranked *hd*PCA data, with the ranking based on *P-*value from low (highly significant) to high (not significant) (Figure 2D). The same principle was applied for directional analysis (Figure 2C) but the ranking of the *hd*PCA data was done from lowest *P-*value with reduction in *hd*PCA signal on rapamycin to lowest p value with increase in *hd*PCA signal on rapamycin, with non-significant data (high *P-*value) in the middle.

#### Gene ontology enrichment analyses

Two sets of hits, *hd*PCA hits showing an increase in their signal in the control condition versus in the presence of metformin and those showing an increase in their signal in the metformin versus the control condition, were investigated for enriched biological processes, molecular functions and cellular compartments using the conditional hypergeometric test of the Bioconductor GOstats package (Falcon and Gentleman, 2007). In these tests, the universe was set to the 3,504 proteins that were tested in the metformin screen and the *P*-value cutoff was set to 0.05. The conditional hypergeometric test performs a GO enrichment analysis that takes into account the relationship between the tested terms in the GO tree structure. This guarantees that the most specific enriched terms will be identified, as the test of enrichment begins to test terms that are located at the bottom of the tree. By going up the tree, child terms that exhibit a significant enrichment will be excluded from the enrichment test of their parents. The GO enrichment analysis generated two lists of enriched biological processes corresponding to the two sets of hits mentioned above, molecular functions, and cellular compartments (Table S4). Subsequently, a visual summary was generated to enriched biological processes by the EnrichmentMap Cytoscape plugin (Merico et al., 2010) in the form of a Cytoscape network (Merico et al., 2010, Smoot et al., 2011). In this network, nodes represent the enriched biological processes that were generated by the GOstats package previously. Nodes are linked by edges if the Jaccard coefficient, representing an overlap measurement, between the linked nodes is higher than 0.25. Thickness of the edges is proportional to the Jaccard coefficient of the linked nodes. Network nodes were arranged by the Cytoscape force directed layout weighted mode. Thus biological processes that are functionally related are closer to each other in space than those that are not. To facilitate the interpretation of the resulting enriched biological processes, the AutoAnnotate Cytoscape plugin (Kucera et al., 2016)was used to cluster biological processes that are significantly related and to label these clusters with relevant terms (Kucera et al., 2016). The default parameters of AutoAnnotate were used. Some labels were renamed to better represent the clustered GO terms and some spatial arrangement was performed to enhance clarity.

#### Diabetes- and cancer-related SNPs versus metformin *hd*PCA

All single-nucleotide polymorphisms associated to type 2 diabetes (T2D) and to all cancers were collected from a manually curated genome-wide association studies (GWAS) database called GWAS Catalog that is provided jointly by the National Human Genome Research Institute (NHGRI) and the European Bioinformatics Institute (EMBL-EBI) (Welter et al., 2014). The collected GWAS data were filtered by two criteria: one applied on the level of the study and the other on the level of SNPs. First, GWA studies should assay SNPs in control versus disease cases. Second, SNP-disease associations should have a *P-*value < 10^−5^. Human genes that contain the collected SNPs were mapped to their homologs in the budding yeast using the Bioconductor biomaRt package and the BioMart database (Durinck et al., 2009). For each type of disease, the set of homologs in the budding yeast of human genes containing SNPs associated with this disease were tested for significant overlap with genes encoding hits of the metformin *hd*PCA by applying the hypergeometric test (Table S5). Only homologs of genes associated with the T2D and with the prostate cancer showed significant overlap with a *P*-value cutoff of 0.05. The detailed lists of these homologs are represented in Tables S6 and S7.

#### Iron limitation genetic data versus metformin *hd*PCA

Genes whose deletion enhanced sensitivity to iron-limited conditions (Jo et al., 2009) were tested for enrichment in the set of *hd*PCA hits with positive signal in the presence of metformin through a hypergeometric test.

### Data and Software Availability

Raw images, raw data, Fiji and R scripts are available upon request.

## Supplemental Information

Document S1. Tables S5–S7

Table S1. hdPCA Hits with a Decrease or Increase in Signal on Rapamycin versus the Control, Related to Figure 2

Table S2. hdPCA Hits with a Decrease or Increase in Signal on Metformin versus the Control, Related to Figure 3

Table S3. Systematic Literature Study of Homodimerization and Protein Activity, Related to Figure 2

Table S4. Enriched GO Biological Processes, Molecular Functions, and Cellular Compartments in hdPCA Hits with an Increase or Decrease in Signal on Metformin versus the Control, Related to Figure 3

Table S8. Raw and Processed Data of the ICP-MS Experiment to Determine the Effect of Metformin on the Concentration of Protein-Bound Iron, Related to Figure 5
