# Supplementary material for: Changes of Cell Biochemical States Are Revealed in Protein Homomeric Complex Dynamics
Source: Cell. 2018 Nov 15;175(5):1418–1429.e9. doi: 10.1016/j.cell.2018.09.050 (PMC6242466; doi:10.1016/j.cell.2018.09.050)
Supplement: Document S1. Tables S5–S7 [file mmc1.pdf]

Cell, Volume *175*

Supplemental Information

Changes of Cell Biochemical States Are Revealed in Protein Homomeric Complex Dynamics

Bram Stynen, Diala Abd-Rabbo, Jacqueline Kowarzyk, Leonor Miller-Fleming, Simran Kaur Aulakh, Philippe Garneau, Markus Ralser, and Stephen W. Michnick

Table S5. Significance of the overlap between metformin hits and homologs in the budding yeast for genes associated to different diseases in *Homo sapiens* (related to Figure 3).

**Disease**	**GWA studies (#)**	**Associated SNPs (#)**	**Mapped *Homo sapiens* genes (#)**	**Homolog ORFs in yeast (#)**	**Tested ORFs in metformin *hd*PCA (#)**	**Common ORFs with metformin *hd*PCA hits (#)**	**Hyper-geometric test (*P*-value)**
Breast cancer	30	135	138	41	26	3	0.937
Breast cancer (early onset)	1	18	11	10	8	1	0.852
Colorectal cancer	20	82	75	22	19	4	0.599
Epithelial ovarian cancer	2	22	54	16	11	3	0.424
Lung cancer	11	27	43	11	9	1	0.883
Ovarian cancer	5	15	44	13	10	2	0.661
Pancreatic cancer	6	48	121	28	20	2	0.946
Prostate cancer	23	142	313	69	42	15	0.021
Type 2 diabetes | Type 2 diabetes and other traits	42	231	185	45	26	11	0.012

Table S6. Common ORFs between metformin hits and homologs in the budding yeast for genes associated with type 2 diabetes in *Homo sapiens* (related to Figure 3).

Yeast ORF	Yeast Name	Homologs in *Homo sapiens*	GWAS # showing disease association	Pubmed ID	Description
YBR164C	*ARL1*	*ARL15*	1	24509480	Soluble GTPase with a role in regulation of membrane traffic
YOL016C	*CMK2*	*CAMK1D*	3	18372903|20862305|22961080	Calmodulin-dependent protein kinase
YGL190C	*CDC55*	*PPP2R2C*	1	19734900	Non-essential regulatory subunit B of protein phosphatase 2A (PP2A)
YOR316C	*COT1*	*SLC30A8*	12	17293876|17460697|17463246|19056611|19401414|17463248|17463249|19734900|20581827|22693455|23945395|24509480	Vacuolar transporter that mediates zinc transport into the vacuole
YHR132C	*ECM14*	*CPA6*	1	25102180	Putative metalloprotease with similarity to zinc carboxypeptidases
YIL097W	*FYV10*	*MAEA*	2	22158537|23945395	Subunit of GID complex
YKL101W	*HSL1*	*HUNK*	1	21490949	Nim1p-related protein kinase
YMR165C	*PAH1*	*LPIN2*	1	21490949	Mg2+-dependent phosphatidate (PA) phosphatase
YER165W	*PAB1*	*RBMS1*	1	20418489	Poly(A) binding protein
YLR403W	*SFP1*	*JAZF1*	3	20581827|24509480|18372903	Regulates transcription of ribosomal protein and biogenesis genes
YIL030C	*SSM4*	*MARCH1*	1	21490949	Ubiquitin-protein ligase involved in ER-associated protein degradation

Table S7. Common ORFs between metformin hits and homologs in the budding yeast for genes associated with prostate cancer in *Homo sapiens* (related to Figure 3).

Yeast ORF	Yeast Name	Homologs in *Homo sapiens*	GWAS # showing disease association	Pubmed ID	Description
YJR057W	*CDC8*	*DTYMK*	1	23535732	Thymidylate and uridylate kinase
YKR034W	*DAL80*	*GATA5*	1	23535732	Negative regulator of genes in multiple nitrogen degradation pathways
YKL002W	*DID4*	*LINC00506 | CHMP2B*	1	19767753	Class E Vps protein of the ESCRT-III complex
YJL110C	*GZF3*	*GATA5*	1	23535732	GATA zinc finger protein
YAL029C	*MYO4*	*MYO6*	1	25217961	Type V myosin motor involved in actin-based transport of cargos
YBR055C	*PRP6*	*PRPF6*	1	23535732	Splicing factor
YGR132C	*PHB1*	*PHB*	1	23535732	Subunit of the prohibitin complex (Phb1p-Phb2p)
YER095W	*RAD51*	*RAD51B*	1	23535732	Strand exchange protein
YJL026W	*RNR2*	*RRM2*	1	23535732	Ribonucleotide-diphosphate reductase (RNR), small subunit
YJL080C	*SCP160*	*HDLBP*	1	23535732	Essential RNA-binding G protein effector of mating response pathway
YLR403W	*SFP1*	*JAZF1*	1	18264096	Regulates transcription of ribosomal protein and biogenesis genes
YDR523C	*SPS1*	*STK25*	1	23535732	Putative protein serine/threonine kinase
YIL030C	*SSM4*	*MARCH8*	1	25217961	Ubiquitin-protein ligase involved in ER-associated protein degradation
YDL185W	*VMA1*	*ATP6V1A*	1	23535732	Subunit A of the V1 peripheral membrane domain of V-ATPase
YFL021W	*GAT1*	*GATA5*	1	23535732	Transcriptional activator of nitrogen catabolite repression genes
